# Enhancing surface drainage mapping in eastern Canada with deep learning applied to LiDAR-derived elevation data

**DOI:** 10.1038/s41598-024-60525-5

**Published:** 2024-05-01

**Authors:** Mathieu F. Bilodeau, Travis J. Esau, Qamar U. Zaman, Brandon Heung, Aitazaz A. Farooque

**Affiliations:** 1 Department of Engineering, Faculty of Agriculture, Dalhousie University, Truro, NS B2N 5E3 Canada; 2 Department of Plant, Food, and Environmental Sciences, Faculty of Agriculture, Dalhousie University, Truro, NS B2N 5E3 Canada; 3 School of Sustainable Design Engineering, University of Prince Edward Island, Charlottetown, PE C1A 4P3 Canada

**Keywords:** Mask RCNN, Deep learning, Dykelands, Remote sensing, Precision agriculture, NOVA Scotia, Climate change, Environmental sciences

## Abstract

Agricultural dykelands in Nova Scotia rely heavily on a surface drainage technique called land forming, which is used to alter the topography of fields to improve drainage. The presence of land-formed fields provides useful information to better understand land utilization on these lands vulnerable to rising sea levels. Current field boundaries delineation and classification methods, such as manual digitalization and traditional segmentation techniques, are labour-intensive and often require manual and time-consuming parameter selection. In recent years, deep learning (DL) techniques, including convolutional neural networks and Mask R-CNN, have shown promising results in object recognition, image classification, and segmentation tasks. However, there is a gap in applying these techniques to detecting surface drainage patterns on agricultural fields. This paper develops and tests a Mask R-CNN model for detecting land-formed fields on agricultural dykelands using LiDAR-derived elevation data. Specifically, our approach focuses on identifying groups of pixels as cohesive objects within the imagery, a method that represents a significant advancement over pixel-by-pixel classification techniques. The DL model developed in this study demonstrated a strong overall performance, with a mean Average Precision (mAP) of 0.89 across Intersection over Union (IoU) thresholds from 0.5 to 0.95, indicating its effectiveness in detecting land-formed fields. Results also revealed that 53% of Nova Scotia’s dykelands are being used for agricultural purposes and approximately 75% (6924 hectares) of these fields were land-formed. By applying deep learning techniques to LiDAR-derived elevation data, this study offers novel insights into surface drainage mapping, enhancing the capability for precise and efficient agricultural land management in regions vulnerable to environmental changes.

## Introduction

Agricultural dykelands in Nova Scotia are an essential component of the agricultural landscape, providing fertile land for cultivation and supporting diverse ecosystems^1^. These low-lying areas have unique drainage characteristics and land formations. Dykeland soils are highly mineralized, high in silt content, and very compact, making them difficult to drain^2^. Low permeability of the soil often makes the fields water-logged due to high precipitation early in the fall^3^. High rainfalls are a consequence of the coastal effect on Nova Scotia’s weather and its geographical vulnerability to hurricanes^4^. During winter, snow and ice prevent the lowering of the water table resulting in saturation of the top soil layers when snow accumulations melt in the spring^3^. These conditions create acute drainage problems on the dykelands, resulting in difficult farming conditions.

In the 1700s, Acadians settlers solved drainage problems by digging ditches that followed the natural slope of the land. In flat areas, they built small excavated channels separated by parallel ditches approximately 20 m apart^5^. The length between the ditch areas would vary depending on the location but could extend up to several hundred meters. On uneven ground, low-lying areas were drained by digging ditches along the path of least resistance and would be connected to the nearest ditch. This often resulted in agricultural dykelands with irregular ditch lengths, uneven surfaces, and small flats. Although efficient for hand and horse work, this arrangement later posed a challenge for modern farm equipment and proper usage of dykelands^5^.

Accurate mapping and characterization of these fields remain a significant challenge due to their irregular field boundaries, non-linear drainage patterns, and variable surface topography. Recent advancements in remote sensing technology—particularly the availability of high-resolution airborne LiDAR data across Canada—offer promising opportunities for improved land-use analysis and agricultural management^6^. Machine learning and deep learning algorithms have gained significant attention for their potential to process and analyze large-scale high-dimensional datasets^7–9^. These techniques have been increasingly applied to various agricultural tasks, such as crop type classification, yield prediction, and field boundary delineation^10–12^. Recent advances in delineating field boundaries using deep learning algorithms have demonstrated remarkable results with an average Intersection over Union (IoU) accuracy score of 0.94^13^. This represents a significant milestone since the precise identification of boundaries within agricultural fields is a critical prerequisite for any meaningful land use analysis—although the full scope of their utility remains to be tested. For instance, field boundaries delineation algorithm can play a significant role for growers since they can use them to optimize implement size, which contributes to a better efficiency in the field management processes. Additionally, field boundaries are crucial in the development of GNSS guidance maps, offering invaluable spatial data for precision farming practices.

Deep learning (DL) is a subfield of machine learning that uses neural networks with multiple layers to model and understand complex patterns in datasets. These characteristics enables computers to make predictions. DL have multiple layers between input and output nodes, granting it distinct advantages which include the ability to learn from data with minimal human intervention, robustness to natural variations in data, and efficient handling of high-dimensional data^14^. DL models are trained using labelled datasets, with each example comprising an input vector and an associated output label. The model learns to map inputs to outputs, adjusting its internal parameters based on the error it made during backpropagation^15^.

These DL models, have demonstrated promising results in various applications, such as underwater archaeology and damage detection in buildings. A study by^16^ provides a compelling example. They demonstrated the effective use of the YOLOv3 architecture, combined with topo-bathymetric data, to detect shipwrecks. Their results exhibited high accuracy, with an F1 score of 0.92 and a precision of 0.90, highlighting the potential of using DL models in underwater archaeology. Similarly, other researchers have applied machine learning techniques to aerial imagery for damage detection^17^. Revealed the efficacy of drone imagery for identifying cracks and structural damage in buildings, achieving a remarkable global accuracy of 0.990. Their approach employed a CNN architecture and integrated guided filtering (GF) to fine-tune predicted outputs. This methodology yielded numerous benefits, including noise reduction, high-level feature supervision, and the incorporation of both multi-scale and multi-level features during the training process. In a different context, Corbane et al.^18^, adopted a deep learning-based framework for extracting human settlements from Sentinel-2 satellite images^18^. Their work highlighted the vast potential of deep learning in remote sensing applications across an extensive geographic extent. The results presented by Corbane and his team underscore the exciting promise of utilizing deep learning techniques in remote sensing to tackle an array of complex tasks.

Convolutional neural networks (CNNs), a subset of deep learning, have also shown impressive results in image classification tasks, such as recognizing patterns and features in remotely sensed images that can be used to accurately identify and map field boundaries^12^. For instance, CNNs can classify land cover types from multi-spectral satellite images by learning distinctive spatial and spectral features. CNNs can also detect specific objects, offering valuable insights for urban planning, environmental monitoring, and disaster management^19^.

Expanding on the capabilities of CNNs, Mask R-CNN has been developed for instance segmentation tasks, providing a pixel-wise classification of objects in an image^20^. Evolving from Faster R-CNN, this technology uses a Region Proposal Network (RPN) to identify potential object-containing regions of interest (RoIs) within an image. These RoIs are then passed through a convolutional network, generating independent feature maps for each RoI^21^. This method facilitates localized feature extraction and leads to more precise mask predictions, yielding binary masks for each object instance. Mask R-CNN has shown potential for automating the mapping of topographic features from digital elevation data, a task traditionally riddled with time-consuming and labour-intensive manual interpretation^22^. While deep learning, CNNs, and Mask R-CNN have revolutionized tasks such as feature extraction, classification, and detection on large geographic extents, their application to the detection of surface drainage characteristics on agricultural fields remains a gap that needs to be addressed.

The primary objective of this research was to develop and test a Mask R-CNN model for detecting land-formed fields on agricultural dykelands using high resolution Digital Elevation Models (DEMs) derived from LiDAR data. By incorporating DEMs and training the model with representative samples of land-formed field, we expect to achieve positive detection of surface drainage patterns on agricultural dykelands. Our technique concentrates on grouping pixels into objects within the images, which differs from methods that classify each pixel separately. This research will contribute to a better understanding of land use, leading to more efficient management practices and policies. This novel research also showcases how other deep learning algorithms, namely field boundary delineation, can be used as the foundation blocks to characterize other land features on large geographic extents, thus further expanding on their potential uses in agricultural studies.

## Materials and methods

### Historical context

#### Land-forming

The first experiment in drainage and reformation of dykelands in Atlantic Canada was initiated in 1922 at the Experimental Farm in Nappan, Nova Scotia. At the time, researchers at the farm had to increase the distance between the ditches to accommodate the trafficability of small motorized farming equipment. This decision resulted in increased cultivated areas and fewer drainage ditches, and they observed little to no impact on the effectiveness of drainage^23^.

This phase of experimentation continued until the 1950s and involved the construction of multiple field sections, each 23 m in width and ranging from 106 to 274 m in length. Shallow depressions between the ditch areas carried off the water, allowing machines to operate in any direction across the land^5^. Although these draining and reforming experiments were an improvement over previous methods, they were designed for the horse age and were still unsuitable for larger farm machines due to limited distances between ditches. As a result, a new dykeland formation and drainage pattern was required to accommodate modern machines and farming practices. This led to a new series of experiments at the Nappan farm involving land forming.

Land forming is a technique used to alter the topography of fields through the mechanical movement of soil to improve surface drainage. This process involves excavating a series of parallel ditches which are then filled in to create hills called “crowns.” The surface water on the crowns then drains off to the two adjoining ditches^3,24^. The first land forming studies began in 1950. These studies aimed to determine the impact that crowning the fields had on drainage and production^25^. These experiments ultimately led to the crowning of dykelands on larger plots of land (> 2 hectares) with larger ditches (45–60 m wide) and lengths exceeding 457 m. This demonstration showed that wide dykeland ditches (< 30 m) provided adequate drainage and were easier to work with heavy farm equipment, as they improved field trafficability. These surface drainage improvements resulted in a longer growing season and increased crop yields^26^.

In 1986, a report titled “Farm Drainage in the Atlantic Provinces” summarized the results from the experiment at the Nappan farm and recommended a distance between open ditches to be between 35 to 60 m with a gradient of 0.1–0.4% to an open collector ditch^27^. These recommendations have been in place for more than three decades and have propagated across the maritime provinces, making it the modern method of land forming agricultural dykelands^28^.

#### Subsurface drainage

Subsurface drainage trials were conducted on Nappan Farm’s dykelands in the 1950s using tile drains, a system of underground pipes removing excess water from the soil. Initially, attempts with four-inch drainage tiles placed in a field ditch and covered with clay proved to be inadequate to remove excess surface water^5^. In 1954, a new trial was conducted on different types of ditches and drains. Results from this experiment showed that the tiled drains were not functioning properly, as water remained on top of the tile drain in several locations. It was determined that the tiles were unobstructed, but the water was unable to percolate through the soil that covered the tile.

Further trials on tile drains were carried out at the Nappan Farm in 1968 on a wet and poorly drained one hectare area bordering the uplands. The area had several springs at the base of the uplands, which posed a significant issue in this field. To address the problem, the tile drains were connected to the springs to eliminate the water seepage at the surface. The field was also sloped to facilitate water movement towards the area over the tile^5^. Observations over the next six years following the installation revealed that the tile functioned well and effectively drained the field. This drainage and land levelling method offers a viable approach to addressing problematic small wetland areas located proximal to uplands, thereby converting them into more extensive fields free of ditches. Additionally, the elimination of ditch maintenance translates to reduced operational costs^29^.

Understanding this historical context makes it possible to infer land usage based on its forming state. Fields with open ditches spaced 35 to 61 m apart were most likely built later than the 1950s and suggest intensive farming activities. Oppositely, the presence of parallel ditches spaced 15 to 23 m apart indicates that older drainage techniques were used before the 1950s and suggest limited agricultural activities due to their inefficiency in carrying large farming equipment. Finally, the presence of un-formed agricultural dykelands and the use of tile drains have also proven to be a viable solution for fields closer to the uplands.

### Study area

**Figure 1:**
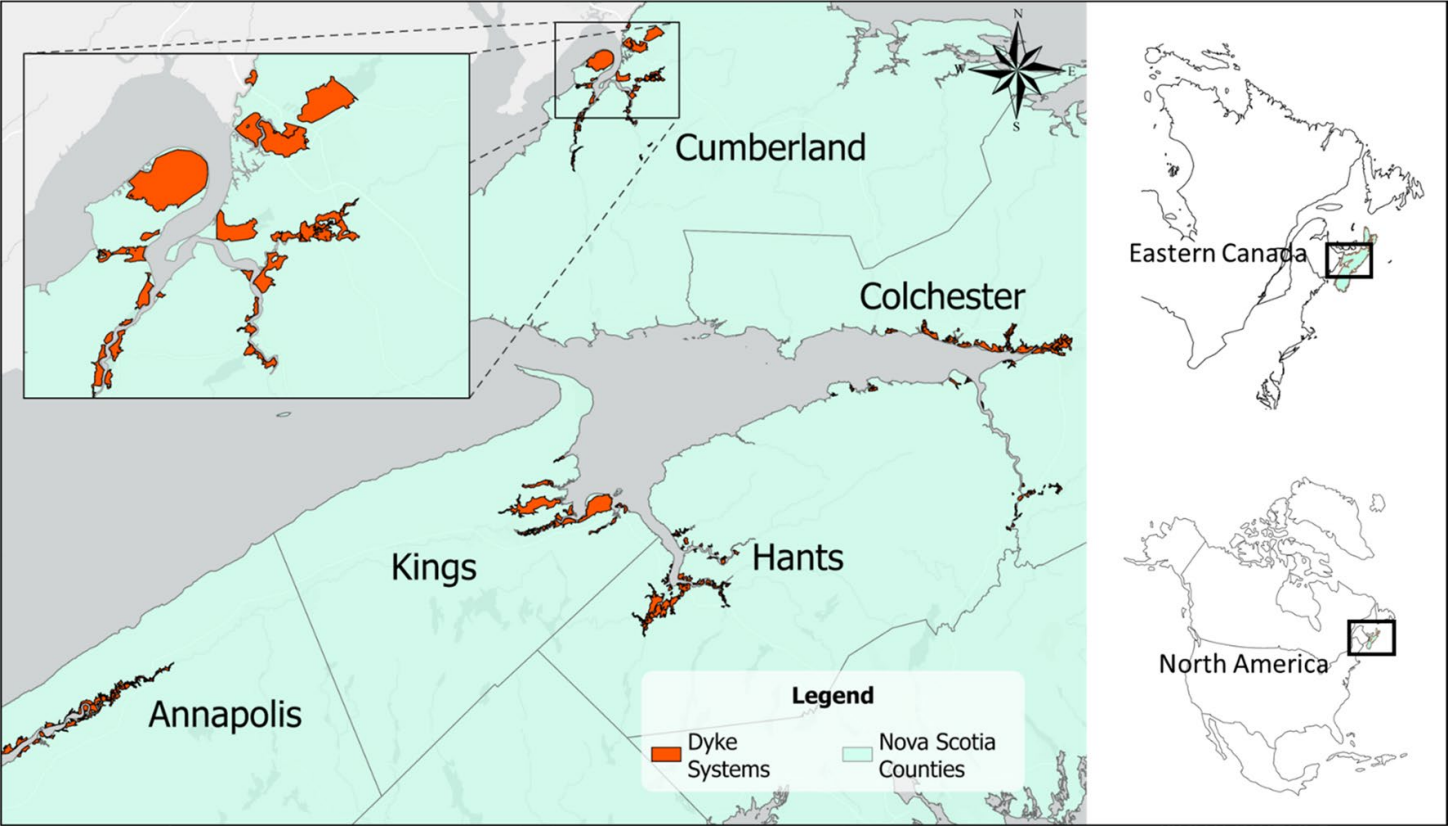
Geographical distribution of Nova Scotia’s dyke systems by counties.

The study area, situated on the East Coast of Canada, encompasses the entirety of Nova Scotia’s dyke system, as defined by the Nova Scotia Agricultural Marshland Conservation Act (Fig. 1). This system spans an impressive 17,401 hectares. Most dykelands in Nova Scotia are situated along the Bay of Fundy, illustrating the interaction of coastal and agricultural landscapes in this part of Canada. These dyke systems spread throughout the counties of Annapolis, Colchester, Cumberland, Digby, Hants, Kings, and Yarmouth (see Supplementary Fig. 1A-G online).

### Datasets

#### Automatic detection of field boundaries

**Figure 2:**
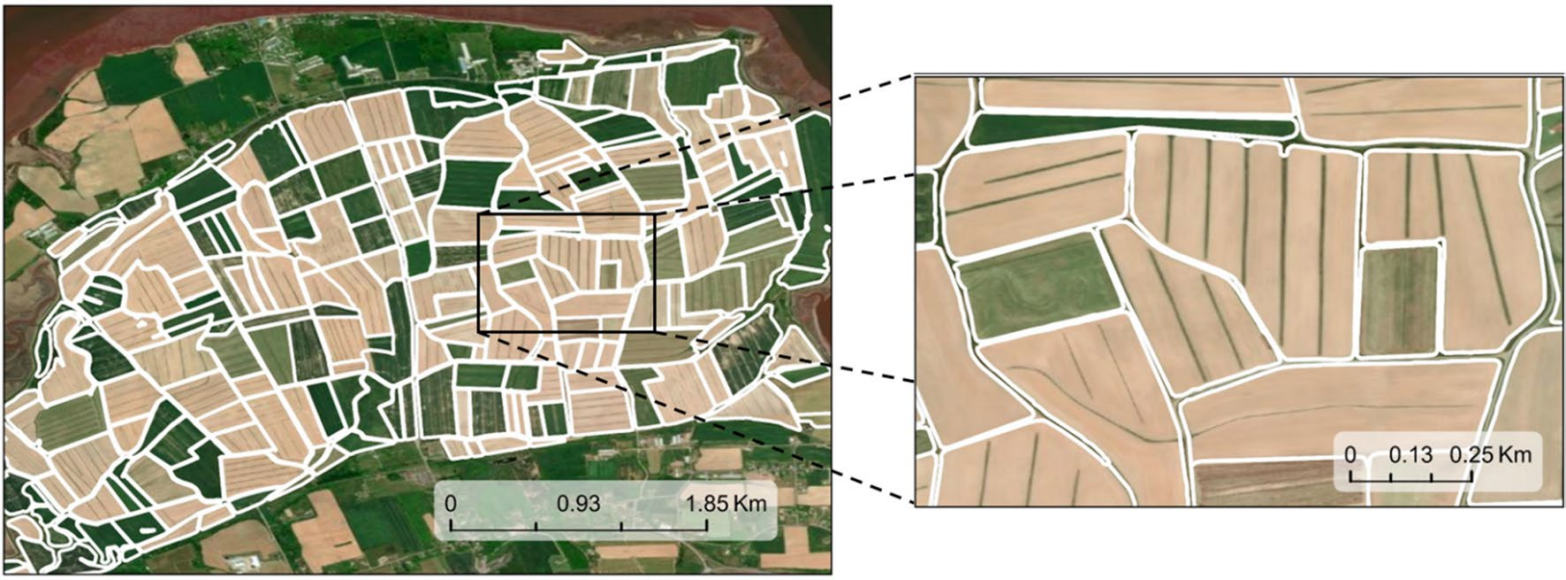
Sample of the field boundaries delineated with the DigiFarm API on the Grand-Pré dyke system.

To distinguish the boundaries of agricultural fields so they could be used to assess the drainage type, DigiFarm (DigiFarm, Inc., Oslo, Norway) provided a dataset of all the field boundaries within the dyke systems. DigiFarm has developed an API that employs a deep neural network model capable of detecting field boundaries from satellite imagery. The model uses high-resolution orthophotos (25 cm spatial resolution) in conjunction with enhanced Sentinel-2 images, upscaled from a 10 m spatial resolution to 1.25 m using a proprietary algorithm (^30^; Fig. 2). DigiFarm’s API was applied to Nova Scotia’s dyke systems, resulting in the digitization of 3421 vector polygons (Table 1). The extent was confined to the limit of the study area and was used to exclude agricultural fields that are not inside the dyke systems. DigiFarm’s deep learning model was exclusively employed for delineating the boundaries of agricultural fields. The outputs from this model were subsequently utilized as reference data (ground truth) to evaluate and enhance the performance of the deep learning model discussed in this manuscript.

**Table 1:** Total number of field boundaries digitized from a deep learning API and manually within Nova Scotia’s dyke system.

County	Digitized features from DigiFarm’s API	Manually digitized features	Total number of digitized features	Total digitized (ha)	Total land (ha)
Annapolis	422	148	570	1491	2296
Colchester	604	28	632	1994	2730
Cumberland	749	36	785	4228	5166
Digby	12	41	53	237	328
Hants	746	28	774	2177	2947
Kings	847	80	927	2925	3642
Yarmouth	41	38	79	210	292
Total	3421	399	3820	13,262	17,401

#### Manual digitization of field boundaries

DigiFarm field boundary delineation API was trained from satellite images of agricultural dykelands and thus struggled to identify boundaries of abandoned agricultural fields due to the presence of dense vegetation. Therefore, the remaining agricultural field boundaries were manually digitized in ArcGIS Pro (ESRI, Redlands, CA, USA). Multitemporal satellite images were used for digitizing missing field boundaries manually. Manual digitization on high-resolution images is a common approach for boundary delineation but has proven to be labour-intensive and time-consuming for large areas^31^.

This digitization process was performed on approximately 3820 hectares of dykelands, representing 399 polygons. To maintain a consistency during the digitization process, three rules of image interpretation were defined^32^. Firstly, tree lines, streams of water, ponds and roads were used as natural boundaries to delineate fields^33^. Secondly, changes in pattern consistency from aerial images were used as indications of new field boundaries. Thirdly, satellite images from the Sentinel-2 and PlanetScope constellations, taken at different dates, were used to reduce ambiguity during the delineation process^34^. The total number of field boundaries delineated from DigiFarm APIs and manually amounted to 13,262 hectares. Roads, railroads, and urban areas, particularly common in Truro, Stewiacke, Windsor, Wolfville, and Annapolis Royal were excluded from the datasets.

#### Digital elevation models

A variety of products derived from LiDAR data were utilized to identify agricultural fields within the dykelands and generate training data for the DL model. LiDAR data are suitable for identifying agricultural fields on dykelands as they can penetrate the dense vegetation canopy cover commonly found in these areas^26,35^.

Leading Edge Geomatics (LEG) was contracted by the province of Nova Scotia to collect LiDAR data as part of an initiative aimed at gathering LiDAR data over a five-year period, with each year focusing on various regions of the province. This research project drew upon data collected during 2019 and 2020. The 2019 phase of data collection covered roughly 35,000 km^2^, encompassing the western and southwestern counties of Nova Scotia, while the 2020 survey targeted approximately 10,000 km^2^ within Cumberland county^36,37^. For the execution of these surveys, LEG utilized a Riegl Q780, Riegl VQ-1560i, and a Riegl VQ-1560ii scanning system. These aerial surveys were complemented by ground verification efforts by a ground team dispatched across the survey sites to collect Real Time Kinematic (RTK) ground control points. These control points were used during the post-processing of the data to validate the precision of the LiDAR surveys. For non-vegetated vertical accuracy (NVA), the average difference between the ground control points and the LiDAR survey was 0.005 m in 2019 and − 0.022 m in 2020, suggesting a very high level of accuracy. The precision details about these surveys are outlined in Table 2. Additional information regarding the methodologies and outcomes of the 2019 and 2020 Nova Scotia LiDAR surveys can be found in the acquisition reports, accessible via the Nova Scotia Geographic Information Services^38^.

**Table 2:** Description of LiDAR data and positional accuracy metrics for surveys conducted in Nova Scotia during the years 2019 and 2020.

Specification	LiDAR dataset	
	2019 collection	2020 collection
Collection dates	May–October	July–September
Sensor	VQ1560i, Q780	VQ1560i, VQ1560ii
Sidelap	20%, 55%	20%
Average post spacing	6 ppsm	6 ppsm
Metric—NVA	Average difference (m): 0.005	Average difference (m): -0.022
	RMSEz (m): 0.055	RMSEz (m): 0.057
	NSSDA (m): 0.107	NSSDA (m): 0.112
Metric—VVA	Average difference (m): 0.078	Average difference (m): 0.008
	RMSEz (m): 0.119	RMSEz (m): 0.046
	NSSDA (m): 0.233	NSSDA (m): 0.091

*ppsm* points per square meter, *NVA* non-vegetated vertical accuracy, *VVA* vegetated vertical accuracy, *RMSEz* vertical root mean square error, *NSSDA* national standard for spatial data accuracy.

To improve the data handling efficiency, datasets were partitioned into 14 km by 14 km scenes encompassing the study area. A total of 20 scenes covering the study area, were employed during the assessment phase (see Supplementary Table 1 online). All LiDAR points were projected onto their respective Universal Transverse Mercator (UTM) zones and, where necessary, adjusted to conform to the Canadian Geodetic Vertical Datum of 2013 (CGVD2013)^39^. From the LiDAR data, a Digital Terrain Model (DTM) was created with a spatial resolution of 1 m. This model included points identified as Ground, Water, and Key-points. The blast2dem tool from the LAStools (rapidlasso GmbH, Gilching, Germany) software suite, was employed for this purpose^36^. This tool facilitated the triangulation of the point cloud, creating an initial Triangulated Irregular Network (TIN) with the longest triangle edges capped at 50 m^37,39^. To address small data-void regions, typically characterized as ‘nodata’ zones, an interpolation approach using the gdal_fillnodata tool was applied, allowing for the fill-in of these gaps by extrapolating from adjacent valid pixels, with the interpolation radius set to a maximum distance of 400 m^39^.

**Figure 3:**
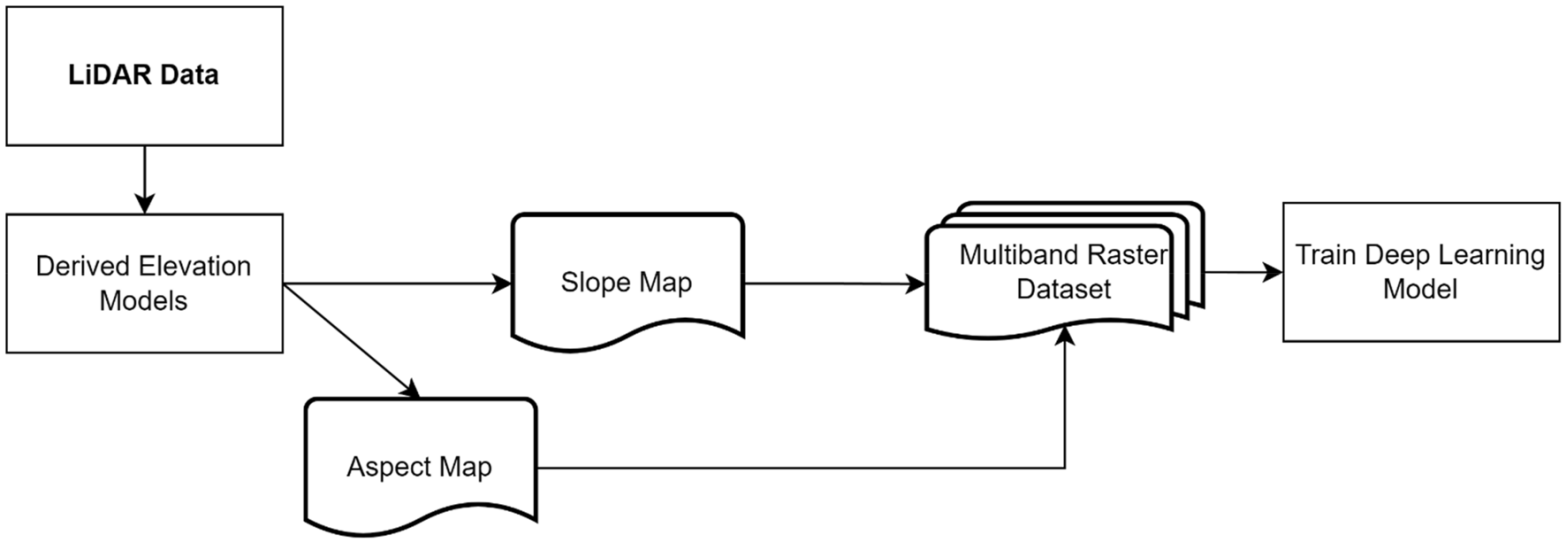
Utilization of LiDAR Data for the generation of multiband raster datasets for the training of a deep learning model.

The aspect and slope functions in ArcGIS Pro were used to create two raster layers from the DEM using the composite band function. The aspect represents the downslope direction of the maximum rate of change in value from each pixel to its neighbouring pixels^40^. It is reflected as the compass direction and symbolized by varying hues. Slope, on the other hand, measures the rate of change in elevation for each DEM pixel. For this study, the slope inclination was calculated using degree values ranging from 0 to 90^41^. Figure 3 represents the general workflow used to generate the training data for the DL model from the LiDAR data.

### Assessment and classification of surface drainage

Fields were evaluated for land forming by two research assistants (RAs) employed by Dalhousie University. RAs were proficient with the use of geographic information systems (GIS) and were trained during workshop sessions on recognition of land-formed fields. Trainings sessions were designed to help RA to be consistent in the image interpretation and expose them to ambiguous scenarios. To minimize inconsistency errors, the same RAs were used during the digitization process and two rules were established during the workshop sessions. Firstly, the classification of land-formed fields should be made only on positively identified fields. Secondly, any ambiguity in relation to the size, shape, texture or height of land-formed dykelands should be classified in a different category for further assessment^42^. This manual assessment method was inspired by Marshall et al^43^, who showed a similar mapping initiative using a crowd-driven manual digitization approach^43^.

**Figure 4:**
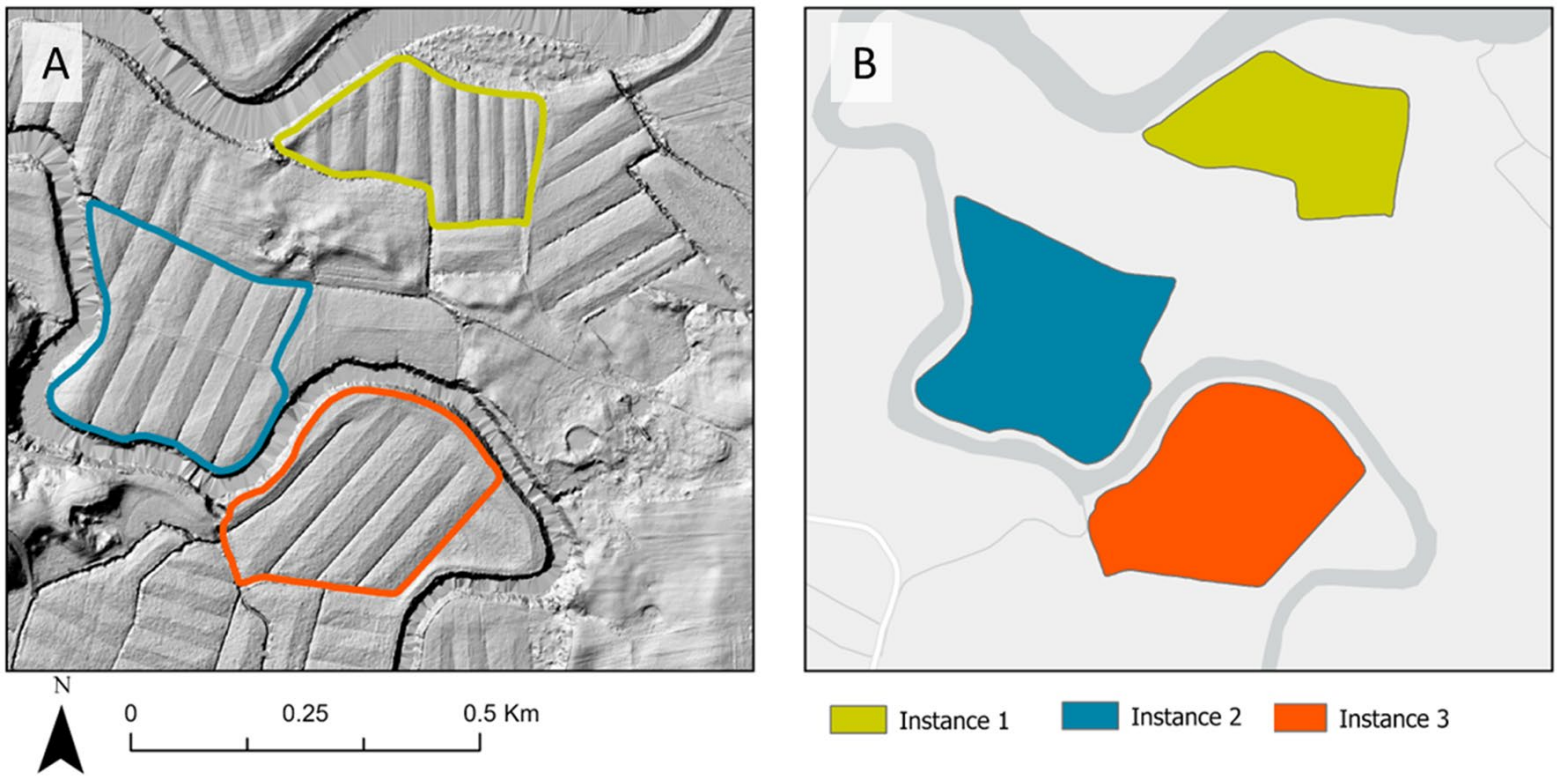
Example of land-formed fields in the study area. Image (**A**) presents a hillshade visualization derived from LiDAR elevation data. Image (**B**) displays the corresponding training mask utilized in developing the deep learning algorithm.

The DEMs were the primary data source for manually assessing if agricultural dykelands were land-formed. This was achieved by creating a new column within the field boundaries vector file’s attribute table and editing the value for each field boundary polygon based on the drainage types. This helped organize the data for each polygon, which was used in later stages for further processing^44^. To facilitate the identification of land-formed fields, DEMs were enhanced during image interpretation using the dynamic range adjustments within ArcGIS Pro (ESRI, Redlands, CA, USA) to stretch the pixel values within the display’s extent^45^. Fields with open ditches spaced 35 to 60 m apart were classified as Land-formed. Figure 4A shows a sample of three agricultural field identified with land-formed features.

Agricultural fields that did not present with signs of land formation were classified as Not Formed or Old Formed/Underutilized. Table 3 contains a brief explanation of each categories used to classify agricultural dykelands and an explanation of the drainage type used to make these determinations.

**Table 3:** Category of fields used to classified agricultural dykelands in Nova Scotia based on their drainage characteristics.

Category	Description	Short description
1	Fields are land-formed with open ditches spaced 35–60 m apart	Land formed
2	Fields are relatively flat with no signs of surface drainage	Not formed
3	Fields are land-formed with ditches spaced 15–23 m apart	Old formed & underutilized

**Figure 5:**
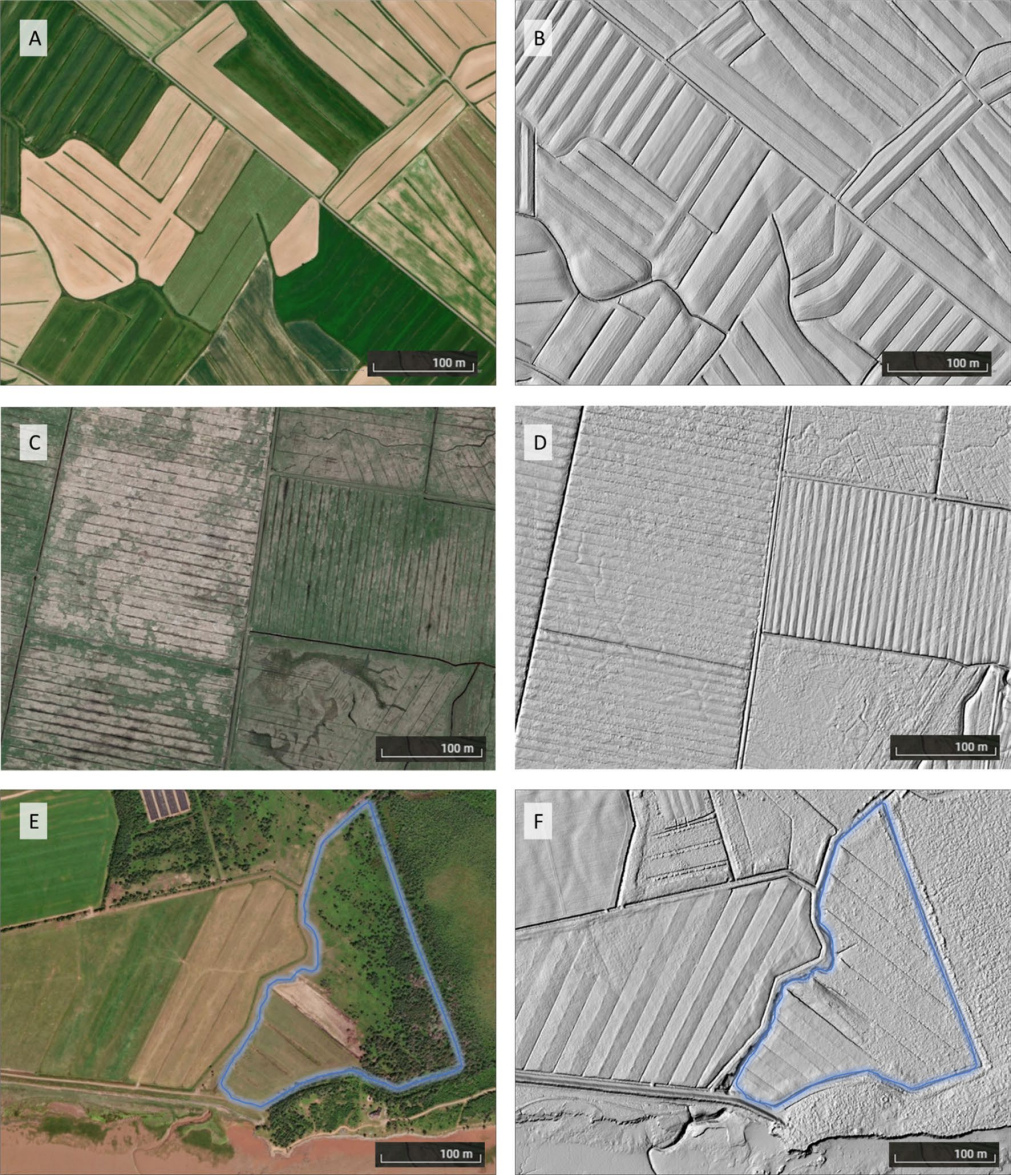
Examples of agricultural dykelands in Nova Scotia, Canada, illustrated through satellite imagery (left) and hillshade visualizations derived from LiDAR elevation data (right): (**A**) & (**B**) show land-formed fields shaped with evenly spaced, open parallel ditches; (**C**) & (**D**) depict a pattern of closely spaced parallel ditches, characteristics of surface drainage techniques used before the 1950s; (**E**) & (**F**) present a land-formed field currently used for agriculture (left side of the image) and adjacent to an underutilized dykelands distinguished by shrubland (highlighted in blue).

Figure 5A,B shows land formed fields presented on satellite images and hillshades generated from the Lidar data datasets. Fields with open ditches 35 to 60 m apart were classified as Land Formed while fields with dales separated by parallel ditches 15–23 m apart were classified as Old Formed/Underutilized (Fig. 5C,D). Additionally, presence of dense shrubland vegetation on land formed fields were used as an indicator of underutilized agricultural lands and were characterized as such (Fig. 5E,F). Remaining agricultural dykelands were classified as Not Formed. Ambiguity in classifying land formed fields were resolved using multi-temporal satellite images, from field visits or from consultations with members of the Nova Scotia Department of Agriculture (NSDA) land protection division.

### Images chip generation

In deep learning techniques that employ convolution, models must be trained on rectangular sections of images rather than individual pixels. This is because convolution effectively captures the spatial relationships between pixels in an image, which cannot be achieved by training on single pixels or isolated components. Therefore, the Export Training Data for Deep Learning tool in ArcGIS Pro was used to generate image chips consisting of 512 × 512 pixel dimensions. These image chips were generated from the aspect and slope raster datasets, and a width and height of 256 pixels in both X and Y directions was applied when exporting them. This created a 50% overlap with adjacent chips.

Using digitized polygons, agricultural fields classified as land-formed were employed as a mask to label features on the image chips (Fig. 4B). Table 4 displays the names of the dyke systems utilized for training, testing, and validating the deep learning model. These areas were non-overlapping and chosen based on their distribution across the province and the number of land-formed fields per dyke system. Dyke system with a high concentration of land-formed fields were prioritized for training the model.

**Table 4:** Number of land-formed fields digitized by dyke system and image chips used to train, test and validate the deep learning model.

Dyke system	County	Total area (ha)	Area Land formed (ha) ground truth	Number of land-formed fields	Image chips with land formed fields	Training & validating (Tr-Val); testing (Test)
Grand Pré	Kings	1206	984	222	856	Tr-Val
Converse	Cumberland	260	153	40	600	Tr-Val
John Lusby	Cumberland	353	223	73	718	Tr-Val
Fort Lawrence	Cumberland	826	337	60	1746	Tr-Val
	Annapolis	2296	486	166	N/A	Test
	Colchester	2730	1381	395	N/A	Test
	Cumberland*	3727	856	192	N/A	Test
	Digby	328	34	13	N/A	Test
	Hants	2947	1374	436	N/A	Test
	Kings*	2436	1013	362	N/A	Test
	Yarmouth	292	81	34	N/A	Test
Total		17,401	6922	1993	3920	

*Excluding dyke systems used to train and validate the model.

Data augmentation has proven to minimize overfitting by increasing the number and variety of training samples^8,46,47^. Hence, augmentation was implemented on the original image chips using angular rotations at 90°, 180°, and 270°, by adjusting parameters within the Export Training Data tool^12,48^. This approach resulted in three additional augmented images per image, aiming to provide the model with more training data that represents the dynamic nature of dykelands fields, which are often found in various orientations, sizes, and shapes. Employing this method, 3920 image chips of digitized land-formed fields were used (80% training, 10% validation, and 10% testing) for training and evaluating the model.

### Model and training

ArcGIS Pro incorporates deep learning capabilities through the utilization of the ArcGIS API for Python. This API is built on top of already established deep learning frameworks such as TensorFlow and PyTorch. This offers a comprehensive interface that facilitates the integration of geospatial data with deep learning models. For object detection tasks, such as those executed with a Mask R-CNN model, ArcGIS Pro employs the deep learning library, ArcGIS Learn^49^. This library streamlines the process of training, fine-tuning, and deploying deep learning models tailored for geospatial data analysis. ArcGIS Learn offers an array of pre-trained models suited to various tasks like object detection. These pre-trained models serve as an initial foundation for transfer learning, enabling the training of models on specific datasets with reduced sample sizes and training durations^50^.

The Train Deep Learning Network tool in ArcGIS Pro was utilized for the process of model training. This tool enables the user to specify parameters such as the maximum number of epochs, the batch size, the chosen deep learning architecture, and the proportion of data used for validation. The training of the model occurs iteratively, leveraging the full dataset for each training cycle. However, given computational constraints, only a random subset of the training dataset was fed into the training algorithm. For this study, the epoch count, which regulates the number of times the dataset was processed during the training phase was set to 500. ArcGIS Pro features a built-in learning rate finder tool that assists in identifying a suitable learning rate by training the model for a few epochs while progressively increasing the learning rate and plotting the training loss against the learning rate.

In this study, a sequential training approach was adopted. Initially, the model was trained using datasets from Kings county for 500 epochs at a learning rate ranging from 0.0001 to 0.00001. Following the effective tuning of this initial training phase, the pre-trained model was used as a starting point to further train it for another 500 epochs on datasets from Cumberland county. The learning rates during the training process for Cumberland county were a slightly lower than those of Kings county, varying between a minimum and maximum boundary of 9 × 10^−5^–9 × 10^−6^. This training approach leveraged the learned features from the Kings county datasets to accelerate and enhance the learning process for the Cumberland county datasets. ResNet-50 was used as the preconfigured neural network backbone and used as the architecture for training the new model. Experimentation with newer backbones, such as ResNet-101 and ResNet-152 were also conducted but yielded lower accuracy. It was theorized that several reasons might explain this. Indeed, while deeper networks can sometimes provide better performance, they also introduce more complexity, which is not always beneficial. In some cases, the additional layers in ResNet-152 may not contribute to the overall performance but increase the chance of issues such as vanishing gradients, exploding gradients, or poor weight initialization^51^. The computational tasks were carried out on a high-performance workstation furnished with a 10-core Intel i9-10900 K processor running at 3.70 GHz, an extensive memory capacity of 128 GB RAM, and a robust GeForce RTX 3090 graphics card with 24 GB of on-board memory.

The evaluation of the model’s performance was made using a metric called ‘loss,’ which quantifies the discrepancy between the model’s predictions and the reference dataset^52^. The aim during training was to minimize this loss, thereby optimizing the model’s predictive accuracy^53^.

### Model prediction

Once the model was trained, it was employed towards detecting land-formed fields within the testing extent of the study area (Table 4). The Detect Objects Using Deep Learning tool in ArcGIS Pro was used to create polygons on detected land-formed fields. Created polygons had a confidence score and class labels associated with each feature. To improve the accuracy of the results, adjustments were made by refining the confidence threshold at 70% and merging overlapping detections using the dissolve tool. The confidence threshold determined the level of confidence required for a delineated land-formed field to be accepted as a successful delineation^50^. An analysis mask polygon of the geographic extent was also used to limit the processing and target only agricultural fields within the dyke system. This adjustment ensured that the final results were more precise and reliable, providing a better representation of the land-formed fields in the study area.

### Validation

The accuracy of the model was calculated using the Compute Accuracy for Object Detection tool in ArcGIS Pro by comparing vector polygons generated from the trained model against the manually classified ground truth data. Five metrics, namely IoU, Average Precision (AP), F1 score, mean Average Precision (mAP), and Precision/Recall curve were used to evaluate the model^54^.

The IoU ratio was used to measure the agreement between the predicted and manually digitized land-formed field. The IoU ratio is the amount of overlap between the vector files generated from the predicted field boundaries and the vector file around the reference data manually digitized. The following formula was used to calculate the IoU:

1 $$IoU=\frac{Area\, of\, Intersection}{Area\, of\, Union}$$

In object classification, a model can predict a positive class or a negative class, and the predictions can be true or false^19^. For example, when detecting the presence of surface drainage on an image, the positive class may be “Land-formed”, while the negative class would be “Not Formed”. A true prediction occurs when the prediction is correct (TP), and a false prediction (FP) occurs when the prediction is incorrect.

Precision, Recall, and F1 score are calculated using True Positives (TP), False Positives (FP), and False Negatives (FN) to provide a comprehensive evaluation of the model’s performance in detecting land-formed agricultural fields. Precision represents the portion of the land-formed fields that were land-formed and is equivalent to 1—commission error. Recall represents the ratio of correctly mapped formed fields relative to the total number of formed fields and is equivalent to 1—omission error^19,54^. The F1 score is the harmonic mean of precision and recall and ranges from 0 to 1 where 1 means highest accuracy. The following formulas were used to calculate the Precision, Recall and F1 score:

2 $$Precision = \frac{TP}{TP+FP}$$

3 $$Recall = \frac{TP}{TP+FN}$$

4 $$F1\, Score = 2 X \frac{Recall\, x \,Precision}{Recall + Precision}$$

## Results

### Mask R-CNN model

**Figure 6:**
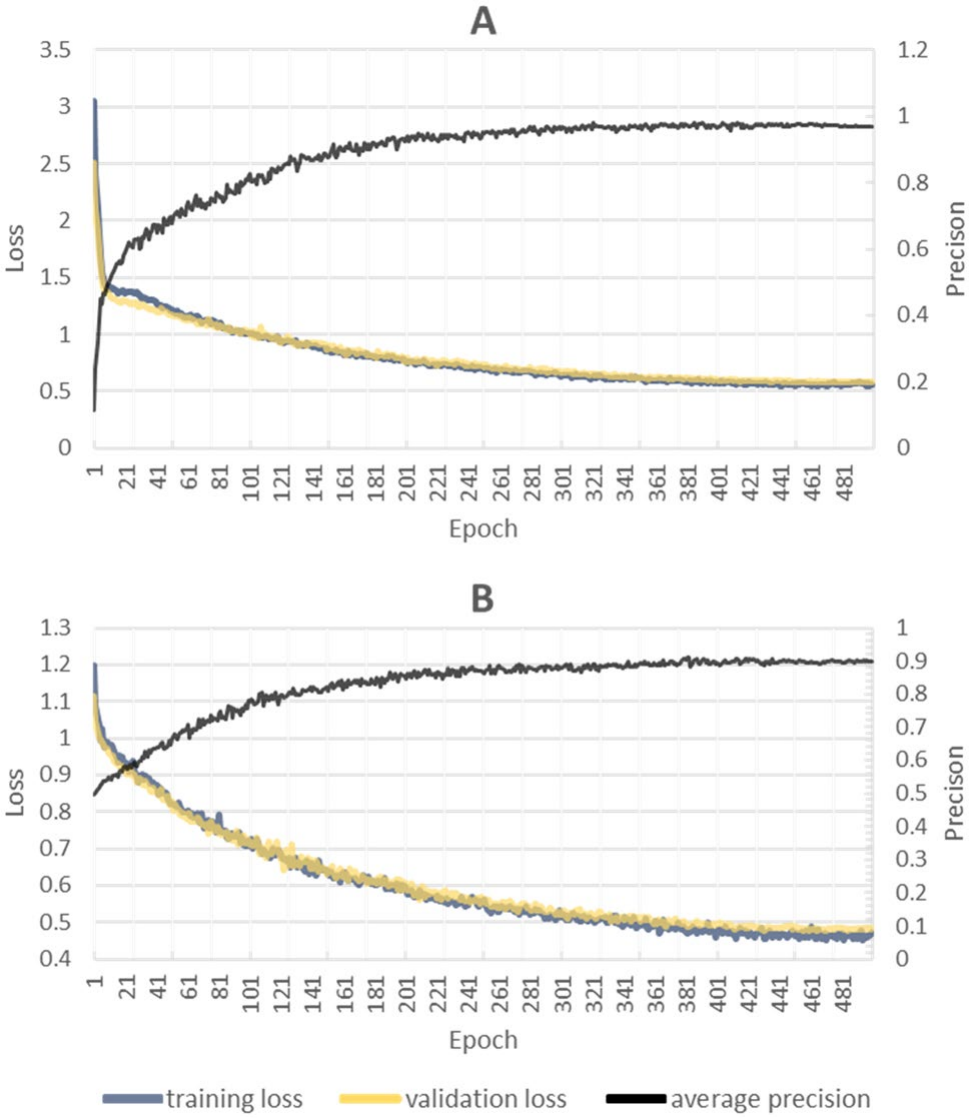
Overall loss values and precision for training and testing data in Kings county (**A**) and Cumberland county (**B**) across all epochs. The training loss is a measure of the difference between the predicted output and the ground truth. Lower values indicate better performance. The validation loss is a measure of how well the model generalizes to unseen data. Lower values indicate better performance.

The training phase for Kings county demonstrated a good level of precision, achieving an average precision score of 0.973. This score, which assesses the model’s aptitude in distinguishing between positive and negative instances of the target class, suggests the model’s performance of higher values indicates better performance. Furthermore, there was a consistent decrease in both training and validation losses over time, indicating the model’s increasing ability to accurately recognize target objects (Fig. 6).

During the initial 200 epochs, we observed an encouraging trend: the model was learning effectively, with losses decreasing and average precision improving consistently. This suggests that the model was continually improving its capacity to correctly classify land-formed fields in the images. However, we also noted that the model’s performance plateaued after the 200th epoch, with validation and training losses stabilizing thereafter.

The training phase for Cumberland county shared a similarly encouraging outcome but achieved a lower level of precision at 0.895. As the epochs increased, we observed a general trend of decreasing training and validation losses, coupled with a consistent improvement in average precision. This combination of factors points to the model effectively learning and enhancing its performance over time. Until the 240th epoch, the model continued to demonstrate improvements. Both losses were decreasing, and the average precision was increasing, indicating no issues with overfitting or underfitting up to this point.

### Validation

Table 5 shows the performance of the evaluation metrics of the DL model for land-formed field detection. The model performed best in Digby county with a precision of 0.792, recall of 0.834, and an F1 score of 0.812, while Yarmouth county exhibited the lowest precision of 0.595. In counties with over 350 accurately mapped fields, namely Kings, Colchester, and Hants, Colchester county stood out by achieving a high model performance with an F1 score of 0.772 at an IoU threshold of 0.5, based on 395 verified fields. Similarly, Hants county demonstrated strong model effectiveness, with an F1 score of 0.754, supported by 436 verified fields. Kings county also showcased efficient model usage, obtaining an F1 score of 0.791 with 362 verified fields.

**Table 5:** Performance Evaluation of the Mask R-CNN deep learning model for the detection of agricultural land-formed fields across various counties in nova scotia: Highlighting the precision, recall, F1 score, and average precision (AP) metrics at multiple intersection over union (IoU) thresholds.

County	IoU (> =)	Precision	Recall	F1 Score	AP	True positive	False positive	False negative	Ground truth land-forme d fields
Annapolis	0.5	0.654	0.772	0.708	0.848	109	57	32	166
	0.75	0.554	0.654	0.600	0.848	92	74	49	166
	0.95	0.401	0.474	0.435	0.848	67	99	74	166
Colchester	0.5	0.743	0.804	0.772	0.918	293	102	72	395
	0.75	0.628	0.675	0.651	0.918	248	147	119	395
	0.95	0.455	0.487	0.470	0.918	180	215	190	395
Cumberland*	0.5	0.760	0.826	0.792	0.897	146	46	31	192
	0.75	0.660	0.710	0.684	0.897	127	65	52	192
	0.95	0.470	0.511	0.490	0.897	90	102	86	192
Digby	0.5	0.792	0.834	0.812	0.955	10	3	2	13
	0.75	0.686	0.734	0.709	0.955	9	4	3	13
	0.95	0.494	0.548	0.520	0.955	6	7	5	13
Hants	0.5	0.729	0.781	0.754	0.934	318	118	89	436
	0.75	0.610	0.654	0.631	0.934	266	170	141	436
	0.95	0.443	0.474	0.458	0.934	193	243	214	436
Kings*	0.5	0.765	0.819	0.791	0.922	277	85	61	362
	0.75	0.665	0.705	0.685	0.922	241	121	101	362
	0.95	0.472	0.508	0.490	0.922	171	191	165	362
Yarmouth	0.5	0.595	0.699	0.642	0.817	20	14	9	34
	0.75	0.457	0.557	0.502	0.817	16	18	12	34
	0.95	0.328	0.403	0.362	0.817	11	23	17	34

mAP @ IoU [0.5: 0.95] @ Land-formed = 0.898, Weighted AP @ IoU [0.5: 0.95] @ Land-formed = 0.912.

*Values calculated excluding dyke systems used to train and validate the model.

Performance generally declined at higher IoU thresholds across all counties, indicating a decrease in model accuracy for more stringent overlap criteria. Across the counties, the AP metric remained consistently high, with a mean AP of 0.898 and a weighted AP of 0.912 for IoU thresholds ranging from 0.5 to 0.95, underscoring the overall robustness of the Mask R-CNN model in detecting land-formed fields.

**Figure 7:**
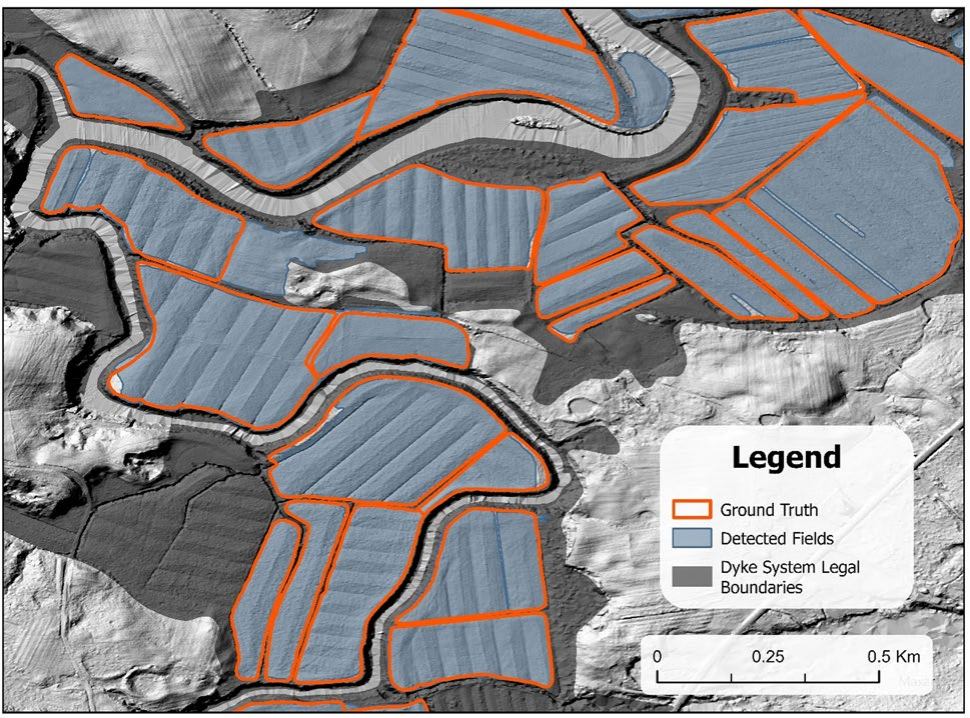
Examples of detected land-formed fields in Hants county, NS. The blue lines represent the objects detected by the Mask-RCNN algorithm, while the red lines indicate the ground truth results that have been manually outlined by researchers for comparison and validation purposes.

Figure 7 illustrates the predicted landform fields juxtaposed against those manually digitized in Hants county. The visual comparison indicates a generally accurate detection of the land-formed fields, with a minimal number of false positives and false negatives. However, there was a noticeable difference in boundary lines, which did not perfectly align with the reference datasets. While the deep learning model proved to be largely successful in its detection capabilities, it encountered some difficulty in identifying land formations along the peripheries of the fields.

**Figure 8:**
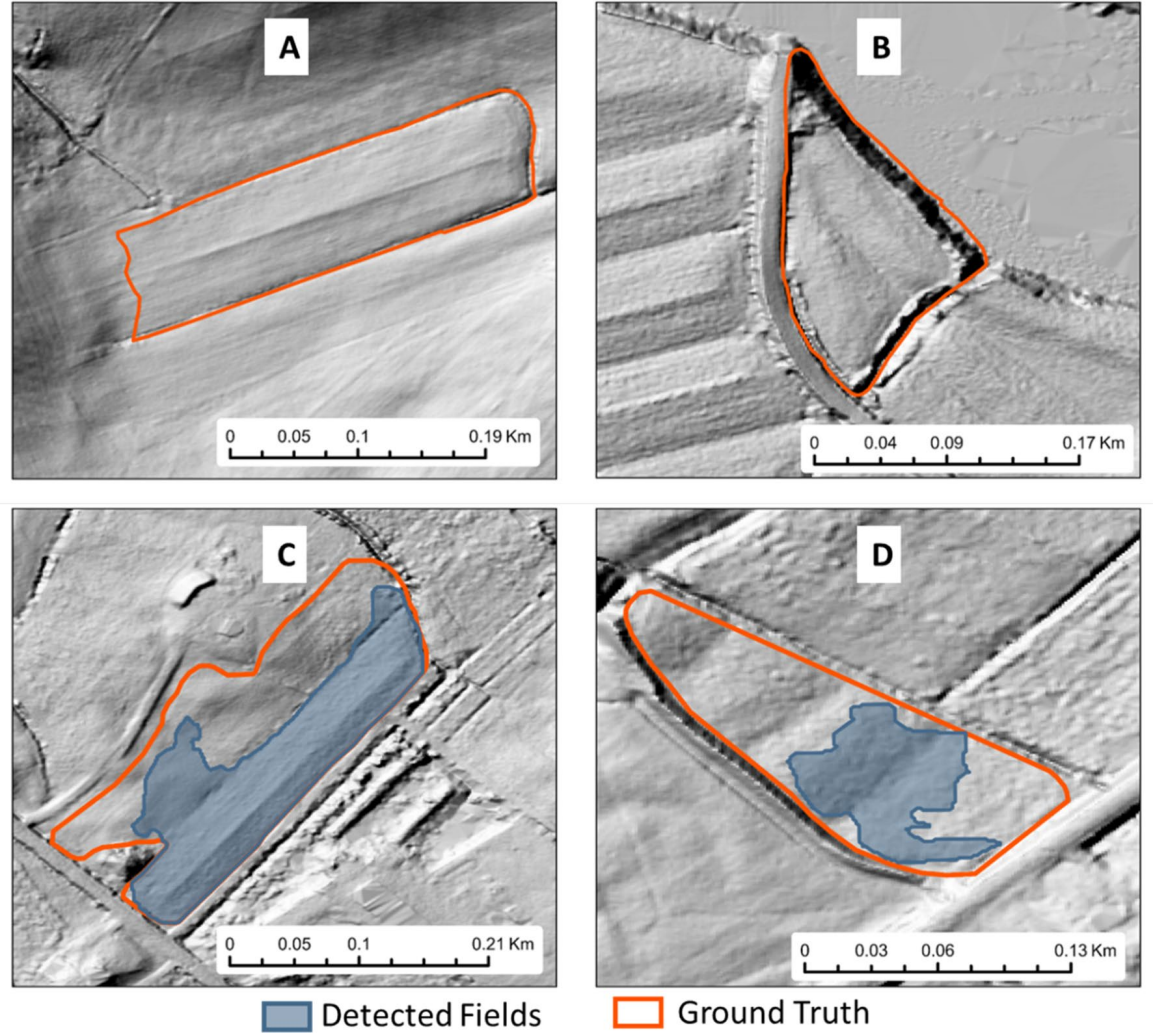
Example of False Negative (**A**), (**B**) and False Positive (**C**), (**D**).

Figure 8 presents common examples of false negativres (FN) and false positives (FP) in relation to land-formed fields. For instance, Fig. 8A demonstrates a shorter section of land-formed fields that was divided by a larger, atypical ditch. Ordinarily, these fields would be formed with an east–west orientation, and this divergence could potentially be the cause of the false negatives. Similarly, Fig. 8B displays a field with an uncharacteristic orientation, namely south-southeast, which was not included in the model’s training datasets and could thus be a contributing factor to a false negative. Figure 8C features a field with an unusual relief pattern that might have confounded the model’s detection capabilities. In Fig. 8D, the upper part of the field showcases a minor slope located between the ditches, a characteristic reminiscent of typical landform fields. This similarity may have confused the model. However, the lower section of the same field displays a varying relief pattern that the model might have struggled to interpret correctly.

### Nova Scotia dykelands

Expanding beyond the deep learning models results, this work also allowed a precise characterization of the size and agricultural use of Nova Scotia’s dykelands. A total of 13,262 hectares were classified across four different categories. The land categories included Land Formed, Not Formed, Old Formed, and Freshwater Marshes/Shrubland. We contrasted the categorized land versus the total amount of land protected by the NSDA. Results revealed that 9272 hectares of dykelands were used for agriculture and almost 75% (6924 hectares) were land-formed while the remaining were not (2347 hectares) (Table 6). Land utilisation also varied significantly between counties, with Cumberland county having the most underutilized dykelands in the province (see Supplementary Table 2 online).

**Table 6:** Number of hectares of dykelands classified by drainage types and agricultural utilisation within the dykelands of Nova Scotia.

Category	Description	Field size (ha)	Used for agriculture	Underutilized
1	Land formed	6924	9,272	
2	Not formed	2347		
3	Old formed	2899		3989
4	Marshes/Shrubland	1090		
Total		13,262		
Total land protected by NSDA		17,401		

## Discussion

### Interpretation and significance of findings

The present study’s findings align with preceding research, which demonstrated the utility of CNNs in feature extraction from elevation models^12,48^. For instance, Maxwell et al., 2020, employed Mask R-CNN for extracting valley fill faces from elevation data, achieving high Precision, Recall, and F1-score exceeding 0.85. Likewise, Zhao et al.^12^, demonstrated the flexibility and adaptability of deep convolutional networks in mapping complex terraces.

Expanding the perspective to a wider scope, the present study reinforces the importance of CNNs in mapping features that possess distinctive spatial, contextual, or textural signatures. This is particularly relevant when these features are not spectrally separable from other classes or features, further solidifying the value of these techniques in the field of remote sensing^55^.

Although the use of an automated approach, as presented in this study, can offer less effort than manual delineation, the practical application of an automated solution is always a semi-automated solution where humans revisit the results and adjust the problems^56^. Consequently, the proposed model should function as a preliminary screening tool, aiding geospatial analysts in refining the classification process. Analysts should validate the model’s outputs, eliminating inconsistencies and outliers. Therefore, the adoption of this deep learning solution would transform the task into one focused on quality assurance rather than traditional manual digitization. This was an important takeaway from the study, where, during the validation phase, fields not classified as land-formed were manually classified into one of the categories listed in Table 3. This process, therefore, had to be completed manually. Although time-consuming and labour-intensive, this process was still more efficient than a complete manual classification. Out of the total 1,598 land-formed fields in Nova Scotia, 1173 were correctly classified by the algorithm at an IoU of 0.5, indicating that 73.40% of the fields did not necessitate manual corrections. For all of the dyke systems, representing 3820 fields, 31.42% of the features required manual intervention to complete a full classification into the four categories.

Advancements in instance segmentation techniques, such as Mask R-CNN, have brought significant enhancements to the field of remote sensing by providing new tools to interpret aerial images versus more traditional techniques, such as Object-Based Image Analysis (OBIA). OBIA operates by segmenting images into objects based on spectral, spatial, and textural characteristics, which are then classified into different categories^57^. However, OBIA often requires manual and time-consuming parameter selection. Alternatively, deep-learning-backed techniques like Mask R-CNN can automatically delineate individual objects within images. These techniques also provide real-time image processing, a vital capability in urgent applications such as disaster response^58^.

Furthermore, Mask R-CNN yields pixel-level object masks, offering more granular and accurate results than OBIA’s typically broader object classifications^57^. Despite the computational and data requirements, continuous advancements in computing infrastructure help mitigate these challenges. Thus, instance segmentation techniques, in compensating for the shortfalls of OBIA, are revolutionizing the efficiency and precision of remote sensing image analysis.

In parallel with these considerations, this study showed that the aspect and slope raster datasets used for preprocessing the training data performed best as it offers several advantages that contribute to better model performance. This representation method captures the intricate morphological variances of terrain, an asset of paramount importance for tasks like landform detection, terrain analysis, and hydrological modeling^59^. This representation enables the model to discern subtler features and variations, thereby potentially increasing its predictive performance.

### Limitations

One of the biggest limitations of the model is that it was trained exclusively on land-formed fields. A more nuanced distinction between old and newly formed fields would have been advantageous, but the scarcity of training areas made this difficult to implement. The potential solution lies in employing newer instance segmentation algorithms or enlarging the dataset to encompass broader study areas. Such an expansion could improve the model’s flexibility, enabling it to differentiate these fields effectively.

The study area stretches 250 km, from the easternmost to the westernmost dyke system. The enormity of this area is not the only challenge; certain systems are disproportionately affected by tidal changes due to the Bay of Fundy’s unique geomorphology. Variations in dyke system, from those directly on the Bay of Fundy to river systems, contribute to diverse land features. Consequently, dykeland fields differ from one dyke system to another. This variability makes it challenging to generate substantial, representative training data needed for training deep learning models. The authors propose that these conditions may partly explain the model’s lower performance with the training data from Cumberland county. This dataset, which accounted for nearly 80% of the total image chips used, was significantly larger than the Kings county dataset. In contrast, the Kings county dataset was limited to the Grand-Pré area, characterized by its homogeneity as most fields are land-formed. Although incorporating the Cumberland county dataset reduced the overall accuracy, it enhanced the model’s robustness and made it more capable of distinguishing land-formed fields across the dykelands.

Finally, despite diligent efforts to minimize errors during the manual classification of land-formed fields, the authors acknowledge the inherent subjectivity of the image interpretation process. Influencing factors may encompass the interpreter’s training and experience, the complexity of the objects being interpreted, and the quality of the images utilized^43^.

## Conclusions

The findings of this study demonstrate the precision of Mask R-CNN in discerning surface drainage characteristics from digital elevation data, achieving a mAP of 0.93 across IoU thresholds ranging from 0.5 to 0.95. This finding highlights the potential of Mask R-CNN as a reliable tool for this purpose. Moreover, boundary delineation algorithms that leverage deep learning models offer a rapid and effective approach to characterizing large geographic areas while offering the possibility to conduct multi-year analysis. By employing these algorithms, we gained valuable insights into drainage patterns and enhance our understanding of land utilization on agricultural dykelands.

However, our model currently falls short in detecting fields that were originally land-formed but have lost their crowned aspect due to poor maintenance. Enhancing this aspect of the model could open up new opportunities for automatically detecting fields in need of reformation, more effectively serving the farming community. It is also worth considering the extension of this training concept to include elevation models generated by drones using photogrammetry-based DEMs. These are generally more cost-effective than their LiDAR-derived counterparts and could provide a valuable resource for refining the proposed algorithm.

Additionally, challenges persist when integrating these advanced algorithms into standard Geographic Information System (GIS) software. These challenges typically stem from the need for custom coding to transform training data into a data type that newer models can process. Consequently, this complexity hinders the ease of incorporating trained models into GIS software, thereby impeding the technology adoption rate in remote sensing and GIS^60^.

Future research avenues could include comprehensively characterizing fields classified as ‘Old Formed.’ This might offer novel insights and deepen our understanding of agricultural dykelands in Atlantic Canada. Moreover, it would be intriguing to examine the applicability of our current model in diverse contexts that implement land-forming techniques. For instance, an immediate area of interest could be the provinces of New Brunswick, which is geographically proximate to Nova Scotia. Indeed, New Brunswick alone boasts over 15,000 hectares of dykelands that could be characterized using our proposed algorithm^61^. Moving beyond regional boundaries, it could be beneficial to extend the application of our model to global locales that utilize land-forming techniques. A notable example is the flat land of the Red River Valley located in Northwest Minnesota, USA^62^. Analyzing such varied geographic regions would undoubtedly strengthen our model’s robustness and generalize its applicability at a wider scale.

Finally, exploring how alternative instance segmentation algorithms, such as the YOLO series could be an interesting avenue of research^63^. This particular algorithm has shown remarkable results in previous studies and could offer valuable insights into better ways of characterizing land features from elevation models^64–66^.

### Supplementary Information

Supplementary Information.

## Data Availability

The datasets used and/or analysed during the current study are available from the corresponding author on reasonable request.
