# Supplementary material for: Enhancing surface drainage mapping in eastern Canada with deep learning applied to LiDAR-derived elevation data
Source: Sci Rep. 2024 May 1;14:10016. doi: 10.1038/s41598-024-60525-5 (PMC11063171; doi:10.1038/s41598-024-60525-5)
Supplement: Supplementary file 1 — Supplementary Information. [file 41598_2024_60525_MOESM1_ESM.docx]

**Enhancing Surface Drainage Mapping in Eastern Canada with Deep Learning Applied to LiDAR-Derived Elevation Data**

**Supplementary Information**

**Mathieu F. Bilodeau^1^, Travis J. Esau^1,*^, Qamar U. Zaman^1^, Brandon Heung^2^ and Aitazaz A. Farooque^3^**

^1^ Department of Engineering, Faculty of Agriculture, Dalhousie University, Truro, NS B2N 5E3, Canada; Mathieu.Bilodeau@dal.ca (M.F.B); qzaman@dal.ca (Q.U.Z.)

^2^ Department of Plant, Food, and Environmental Sciences, Faculty of Agriculture, Dalhousie University, Truro, NS B2N 5E3, Canada; brandon.heung@dal.ca

^3^ School of Sustainable Design Engineering, University of Prince Edward Island, Charlottetown, PE C1A 4P3, Canada; afarooque@upei.ca

* Correspondence: tesau@dal.ca


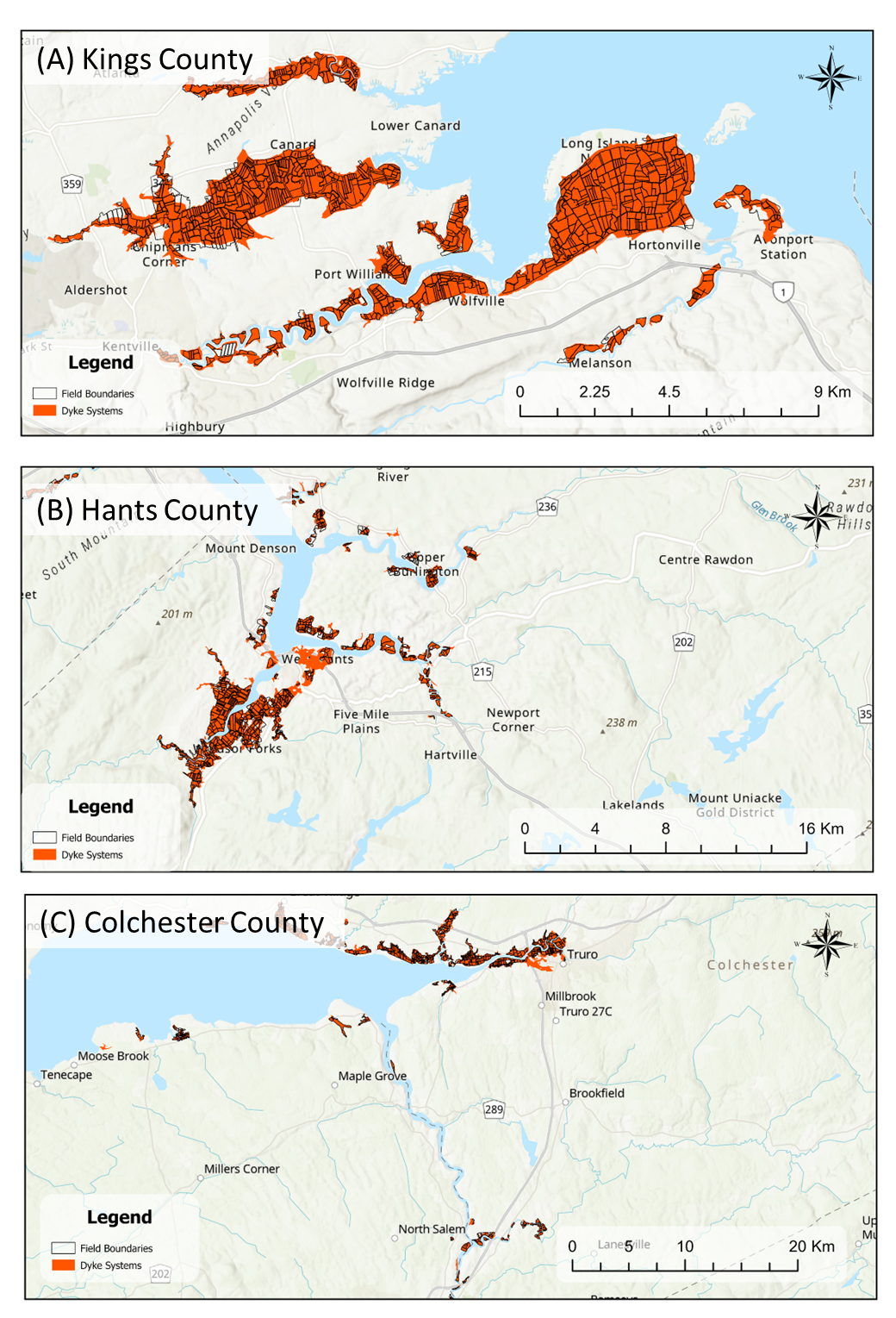


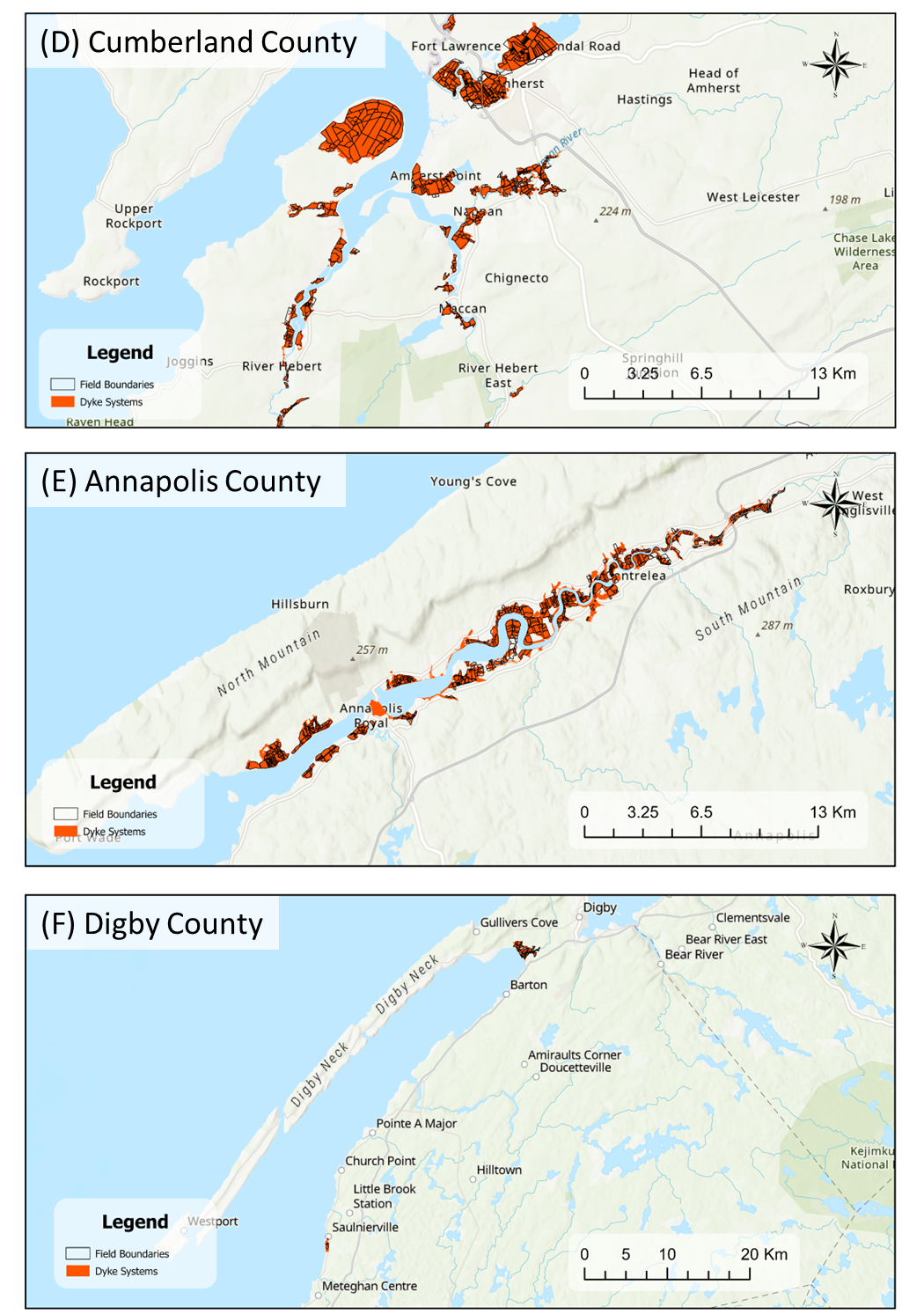


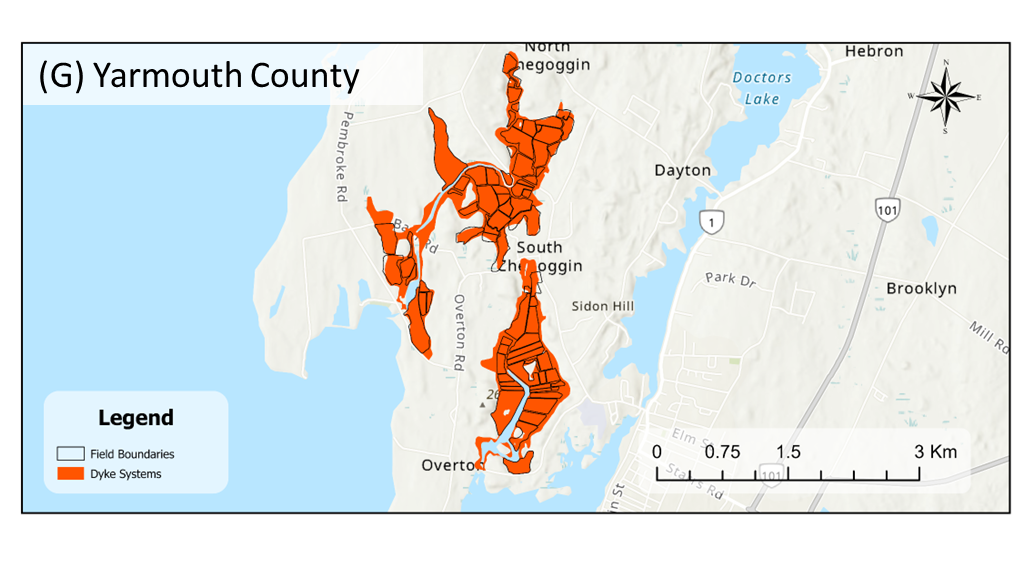


**Supplementary Figure 1.** Geographic mapping of field boundaries and dyke systems across seven counties in Nova Scotia, (A) Kings county, (B) Hants county, (C) Colchester county, (D) Cumberland county, (E) Annapolis county and (F) Digby county.

**Supplementary Table 1.** Inventory of Digital Terrain Model (DTM) tiles for Nova Scotia used to generate aspect and slope maps. These maps were subsequently used to train a deep learning model that can be used to detect surface drainage features. The resource URLs for each DTM tile are constructed by appending the tile-specific path to a base URL, providing public access to the files hosted on the Open Government of Canada online portal.

| **Map Tile Name** | **Project** | **Resource URL** |
| --- | --- | --- |
| 1m_utm20_w_10_99 | NSDNR_2019_2 | [Base URL + "/NS/NSDNR_2019_2/utm20/dtm_1m_utm20_w_10_99.tif"](https://ftp.maps.canada.ca/pub/elevation/dem_mne/highresolution_hauteresolution/dtm_mnt/1m/NS/NSDNR_2019_2/utm20/dtm_1m_utm20_w_10_99.tif) |
| 1m_utm20_w_8_98 | NSDNR_2019_3 | [Base URL + "/NS/NSDNR_2019_3/utm20/dtm_1m_utm20_w_8_98.tif"](https://ftp.maps.canada.ca/pub/elevation/dem_mne/highresolution_hauteresolution/dtm_mnt/1m/NS/NSDNR_2019_3/utm20/dtm_1m_utm20_w_8_98.tif) |
| 1m_utm20_w_9_98 | NSDNR_2019_2 | [Base URL + "/NS/NSDNR_2019_2/utm20/dtm_1m_utm20_w_9_98.tif"](https://ftp.maps.canada.ca/pub/elevation/dem_mne/highresolution_hauteresolution/dtm_mnt/1m/NS/NSDNR_2019_2/utm20/dtm_1m_utm20_w_9_98.tif) |
| 1m_utm20_w_8_99 | NSDNR_2019_3 | [Base URL + "/NS/NSDNR_2019_3/utm20/dtm_1m_utm20_w_8_99.tif"](https://ftp.maps.canada.ca/pub/elevation/dem_mne/highresolution_hauteresolution/dtm_mnt/1m/NS/NSDNR_2019_3/utm20/dtm_1m_utm20_w_8_99.tif) |
| 1m_utm20_w_8_99 | NSDNR_2019_2 | [Base URL + "/NS/NSDNR_2019_2/utm20/dtm_1m_utm20_w_8_99.tif"](https://ftp.maps.canada.ca/pub/elevation/dem_mne/highresolution_hauteresolution/dtm_mnt/1m/NS/NSDNR_2019_2/utm20/dtm_1m_utm20_w_8_99.tif) |
| 1m_utm20_w_2_102 | NSDNR_2019_3 | [Base URL + "/NS/NSDNR_2019_3/utm20/dtm_1m_utm20_w_2_102.tif"](https://ftp.maps.canada.ca/pub/elevation/dem_mne/highresolution_hauteresolution/dtm_mnt/1m/NS/NSDNR_2019_3/utm20/dtm_1m_utm20_w_2_102.tif) |
| 1m_utm20_w_4_102 | NSDNR_2019_3 | [Base URL + "/NS/NSDNR_2019_3/utm20/dtm_1m_utm20_w_4_102.tif"](https://ftp.maps.canada.ca/pub/elevation/dem_mne/highresolution_hauteresolution/dtm_mnt/1m/NS/NSDNR_2019_3/utm20/dtm_1m_utm20_w_4_102.tif) |
| 1m_utm20_w_3_100 | NSDNR_2019_3 | [Base URL + "/NS/NSDNR_2019_3/utm20/dtm_1m_utm20_w_3_100.tif"](https://ftp.maps.canada.ca/pub/elevation/dem_mne/highresolution_hauteresolution/dtm_mnt/1m/NS/NSDNR_2019_3/utm20/dtm_1m_utm20_w_3_100.tif) |
| 1m_utm20_w_6_103 | NSDNR_2019_3 | [Base URL + "/NS/NSDNR_2019_3/utm20/dtm_1m_utm20_w_6_103.tif"](https://ftp.maps.canada.ca/pub/elevation/dem_mne/highresolution_hauteresolution/dtm_mnt/1m/NS/NSDNR_2019_3/utm20/dtm_1m_utm20_w_6_103.tif) |
| 1m_utm20_w_10_107 | NSDNR_2020 | [Base URL + "/NS/NSDNR_2020/utm20/dtm_1m_utm20_w_10_107.tif"](https://ftp.maps.canada.ca/pub/elevation/dem_mne/highresolution_hauteresolution/dtm_mnt/1m/NS/NSDNR_2020/utm20/dtm_1m_utm20_w_10_107.tif) |
| 1m_utm20_w_9_106 | NSDNR_2020 | [Base URL + "/NS/NSDNR_2020/utm20/dtm_1m_utm20_w_9_106.tif"](https://ftp.maps.canada.ca/pub/elevation/dem_mne/highresolution_hauteresolution/dtm_mnt/1m/NS/NSDNR_2020/utm20/dtm_1m_utm20_w_9_106.tif) |
| 1m_utm20_w_9_108 | NSDNR_2020 | [Base URL + "/NS/NSDNR_2020/utm20/dtm_1m_utm20_w_9_108.tif"](https://ftp.maps.canada.ca/pub/elevation/dem_mne/highresolution_hauteresolution/dtm_mnt/1m/NS/NSDNR_2020/utm20/dtm_1m_utm20_w_9_108.tif) |
| 1m_utm20_w_14_102 | NSDNR_2020 | [Base URL + "/NS/NSDNR_2020/utm20/dtm_1m_utm20_w_14_102.tif"](https://ftp.maps.canada.ca/pub/elevation/dem_mne/highresolution_hauteresolution/dtm_mnt/1m/NS/NSDNR_2020/utm20/dtm_1m_utm20_w_14_102.tif) |
| 1m_utm19_e_28_96 | NSDNR_2019_1 | [Base URL + "/NSDNR_2019_1/utm19/dtm_1m_utm19_e_28_96.tif"](https://ftp.maps.canada.ca/pub/elevation/dem_mne/highresolution_hauteresolution/dtm_mnt/1m/NS/NSDNR_2019_1/utm19/dtm_1m_utm19_e_28_96.tif) |
| 1m_utm19_e_30_97 | NSDNR_2019_1 | [Base URL + "/NS/NSDNR_2019_1/utm19/dtm_1m_utm19_e_30_97.tif"](https://ftp.maps.canada.ca/pub/elevation/dem_mne/highresolution_hauteresolution/dtm_mnt/1m/NS/NSDNR_2019_1/utm19/dtm_1m_utm19_e_30_97.tif) |
| 1m_utm19_e_27_95 | NSDNR_2019_1 | [Base URL + "/NS/NSDNR_2019_1/utm19/dtm_1m_utm19_e_27_95.tif"](https://ftp.maps.canada.ca/pub/elevation/dem_mne/highresolution_hauteresolution/dtm_mnt/1m/NS/NSDNR_2019_1/utm19/dtm_1m_utm19_e_27_95.tif) |
| 1m_utm19_e_25_93 | NSDNR_2019_1 | [Base URL + "/NS/NSDNR_2019_1/utm19/dtm_1m_utm19_e_25_93.tif"](https://ftp.maps.canada.ca/pub/elevation/dem_mne/highresolution_hauteresolution/dtm_mnt/1m/NS/NSDNR_2019_1/utm19/dtm_1m_utm19_e_25_93.tif) |
| 1m_utm19_e_22_86 | NSDNR_2019_1 | [Base URL + "/NS/NSDNR_2019_1/utm19/dtm_1m_utm19_e_22_86.tif"](https://ftp.maps.canada.ca/pub/elevation/dem_mne/highresolution_hauteresolution/dtm_mnt/1m/NS/NSDNR_2019_1/utm19/dtm_1m_utm19_e_22_86.tif) |
| 1m_utm20_w_11_99 | NSDNR_2020 | [Base URL + "/NS/NSDNR_2020/utm20/dtm_1m_utm20_w_11_99.tif"](https://ftp.maps.canada.ca/pub/elevation/dem_mne/highresolution_hauteresolution/dtm_mnt/1m/NS/NSDNR_2020/utm20/dtm_1m_utm20_w_11_99.tif) |
| 1m_utm20_w_11_100 | NSDNR_2020 | [Base URL + "/NS/NSDNR_2020/utm20/dtm_1m_utm20_w_11_100.tif"](https://ftp.maps.canada.ca/pub/elevation/dem_mne/highresolution_hauteresolution/dtm_mnt/1m/NS/NSDNR_2020/utm20/dtm_1m_utm20_w_11_100.tif) |
| Base URL: "https://ftp.maps.canada.ca/pub/elevation/dem_mne/highresolution_hauteresolution/dtm_mnt/1m" | | |

**Supplementary Table 2.** Number of hectares of dykelands classified by drainage types and agricultural utilisation, divided by counties within the dykelands of Nova Scotia.

| **County** | **Description** | **Field Size (ha)** | **Used for Agriculture (ha)** | **Underutilized (ha)** |
| --- | --- | --- | --- | --- |
| Annapolis | Land Formed | 486.03 | 862.79 |  |
|  | Not Formed | 376.76 |  |  |
|  | Old Formed | 478.95 |  | 629.20 |
|  | Marshes/Shrubland | 150.25 |  |  |
| Colchester | Land Formed | 1381.25 | 1811.79 |  |
|  | Not Formed | 430.54 |  |  |
|  | Old Formed | 65.44 |  | 182.41 |
|  | Marshes/Shrubland | 116.97 |  |  |
| Cumberland | Land Formed | 1569.42 | 1912.89 |  |
|  | Not Formed | 343.47 |  |  |
|  | Old Formed | 1965.04 |  | 2311.41 |
|  | Marshes/Shrubland | 346.37 |  |  |
| Digby | Land Formed | 34.22 | 37.54 |  |
|  | Not Formed | 3.32 |  |  |
|  | Old Formed | 127.02 |  | 200.36 |
|  | Marshes/Shrubland | 73.34 |  |  |
| Hants | Land Formed | 1374.25 | 1948.26 |  |
|  | Not Formed | 574.01 |  |  |
|  | Old Formed | 87.58 |  | 229.43 |
|  | Marshes/Shrubland | 141.86 |  |  |
| Kings | Land Formed | 1997.63 | 2598.75 |  |
|  | Not Formed | 601.11 |  |  |
|  | Old Formed | 160.67 |  | 327.13 |
|  | Marshes/Shrubland | 166.45 |  |  |
| Yarmouth | Land Formed | 81.71 | 100.22 |  |
|  | Not Formed | 18.51 |  |  |
|  | Old Formed | 14.93 |  | 109.87 |
|  | Marshes/Shrubland | 94.94 |  |  |
| **Total** |  | 13262.04 | 9272.23 | 3989.81 |
